# Allelotypes of lung adenocarcinomas featuring *ALK* fusion demonstrate fewer onco- and suppressor gene changes

**DOI:** 10.1186/1471-2407-13-8

**Published:** 2013-01-05

**Authors:** Hironori Ninomiya, Motohiro Kato, Masashi Sanada, Kengo Takeuchi, Kentaro Inamura, Noriko Motoi, Hiroko Nagano, Kimie Nomura, Yukinori Sakao, Sakae Okumura, Hiroyuki Mano, Seishi Ogawa, Yuichi Ishikawa

**Affiliations:** 1 Division of Pathology, The Cancer Institute, Ariake 3-8-31, Koutou-ku, 135-8550, Tokyo, Japan; 2 Pathology Project for Molecular Targets, The Cancer Institute, Ariake 3-8-31, Koutou-ku, 135-8550, Tokyo, Japan; 3 Thoracic Oncology Center, Cancer Institute Hospital, Japanese Foundation for Cancer Research, Ariake 3-8-31, Koutou-ku, 135-8550, Tokyo, Japan; 4 Cancer Genomics Project, The University of Tokyo, Hongo 7-3-1, Bunkyo-ku, 113-8656, Tokyo, Japan; 5 Department of Pediatrics, The University of Tokyo, Hongo 7-3-1, Bunkyo-ku, 113-8656, Tokyo, Japan; 6 Department of Medical Genomics, Graduate School of Medicine, The University of Tokyo, Hongo 7-3-1, Bunkyo-ku, 113-8656, Tokyo, Japan; 7 Division of Functional Genomics, Jichi Medical University, 329-0498, Tochigi, Japan

**Keywords:** Lung adenocarcinoma, *ALK* fusion, SNP array, Allelotype, Copy number

## Abstract

**Background:**

A subset of lung adenocarcinomas harboring an *EML4-ALK* fusion gene resulting in dominant oncogenic activity has emerged as a target for specific therapy. *EML4-ALK* fusion confers a characteristic histology and is detected more frequently in never or light smokers and younger patients.

**Methods:**

To gain insights into etiology and carcinogenic mechanisms we conducted analyses to compare allelotypes of 35 *ALK* fusion-positive and 95 -negative tumours using single nucleotide polymorphism (SNP) arrays and especially designed software which enabled precise global genomic profiling.

**Results:**

Overall aberration numbers (gains + losses) of chromosomal alterations were 8.42 and 9.56 in tumours with and without *ALK* fusion, respectively, the difference not being statistically significant, although patterns of gain and loss were distinct. Interestingly, among selected genomic regions, oncogene-related examples such as 1p34.3(*MYCL1*), 7q11.2(*EGFR*), 7p21.1, 8q24.21(*MYC*), 16p13.3, 17q12(*ERBB2*) and 17q25.1 showed significantly less gain. Also, changes in tumour suppressor gene-related regions, such as 9p21.3 (*CDKN2A*) 9p23-24.1 (*PTPRD*), 13q14.2 (*RB1*), were significantly fewer in tumours with *ALK* fusion.

**Conclusion:**

Global genomic comparison with SNP arrays showed tumours with *ALK* fusion to have fewer alterations in oncogenes and suppressor genes despite a similar overall aberration frequency, suggesting very strong oncogenic potency of *ALK* activation by gene fusion.

## Background

The adenocarcinoma is the most common form of lung cancer worldwide, different subsets having specific genetic backgrounds of great importance for molecular-targeted therapy. For example, somatic mutations of the epidermal growth factor receptor (*EGFR*) are especially prevalent in adenocarcinomas among never smokers, females, and those with Asian ethnicity [1]. On the other hand, *KRAS* mutations are associated with the smoking habit [2] and the two tend to be mutually exclusive. Recently, Soda et al. found a novel fusion gene, *EML4-ALK*, arising from an inversion on the short arm of chromosome 2 in non-small cell lung carcinomas [3]. *ALK* fusion is a unique example of tyrosine kinase activation by structural chromosome rearrangement [4].

*EML4-ALK* fusion is a powerful driving molecular event by itself. The chimeric protein permits ligand-independent dimerization and constitutive activation of *ALK*, resulting in dominant oncogenic activity. Multiple fusion variants of *EML4-ALK* and notable clinicopathological characteristics of fusion positive tumours have been revealed [5-9]. Since the tyrosine kinase is involved and activated by gene fusion, this type of malignancy has emerged as a target for anti-tyrosine kinase therapy [4,10-12].

We have revealed that *ALK* fusion-positive tumours constituted a particular subset in lung adenocarcinomas in terms of clinical characteristics, histology and etiology, as well as molecular changes [7,8]. It is of great interest to assess global genomic alterations to provide deep insight into their genesis, especially considering these tumours arise in non- or light smokers. Single nucleotide polymorphism (SNP) microarray analysis enables precise high-throughput detection of genomic copy number alterations, gains and losses in the genome contributing to carcinogenesis [13] with gene expression varying consistently with DNA copy number changes [14,15]. We therefore conducted of the present genomic profiling of lung adenocarcinomas with and without *ALK* fusion.

## Methods

### Patient population and specimens

A series of 130 cases of lung adenocarcinomas, 35 with *EML4-ALK* or *KIF5B-ALK* fusion and 95 cases without, were enrolled in this study. From 1998 to 2008, 1,086 primary lung adenocarcinomas were surgically resected at Thoracic Surgery Division, the Cancer Institute Hospital, Japanese Foundation for Cancer Research (JFCR), Tokyo. All cases were screened as to ALK expression by immunohistochemistry using the iAEP method [6] and for positive cases subsequent RT-PCR and FISH analysis were performed, as previously described [5,6,16]. Among them, sufficient amounts and quality of fresh tumour material were available for 35 cases. Fusion gene variants are listed in Addtional file 1: Table S1. V3 constituted the largest proportion, 31% (11/35), having a breakpoint at exon 20 of *EML4*. A rare variant, *KIF5B-ALK* fusion, was detected in two cases. There was no correlation with fusion variant and pathological subtypes (data not shown). The 95 cases without *ALK* fusion were randomly selected from 730 surgically resected adenocarcinomas from 1995 to 2003 at the same hospital. Tissue specimens were snap-frozen in liquid nitrogen, typically within 20 minutes after resection, and stored at −80°C until use. Genomic DNA was extracted by standard proteinase K digestion and the phenol-chloroform method. To confirm if specimens used for analysis in this study contained a significant amount of tumour cells, typically 50% or more, a neighboring surface was examined histologically with frozen sections. This study was approved by the institutional review board of the JFCR.

### Mutation analysis of EGFR, KRAS and TP53

For *EGFR* mutation analysis, exons 18 to 21 were amplified by PCR with specific oligo-primers. For point mutations in exon 18, PCR products were directly sequenced. Fragment analysis was performed for exons 19 and 20 deletions and insertion mutations. The presence of one point mutation in exon 21 was detected by genotyping analysis. To examine *TP53* mutations, direct sequencing from exons 5 to 10 was carried out. For *KRAS* mutation analysis, codons 12, 13 and 61 were examined by direct sequencing. Primers and detailed procedures were as described previously [17].

### Histological diagnosis and clinical staging

Histological diagnosis was made on the basis of World Health Organization (WHO) classification [18] by expe-rienced pathologists (N.M. and Y.I.). Pathological staging was based on the AJCC/UICC staging manual of lung cancer [19]. Differentiation grading of adenocarcinoma was determined essentially according to the Japan Lung Cancer Society criteria as illustrated previously [20]. Briefly, well-differentiated (w/d) tumors are composed chiefly of glands lined by, or of papilla covered by, one-layered tumor cells. Also, Adenocarcinoma in situ (AIS) is included in this category. Moderately differentiated (m/d) lesions comprise glands showing a cribriform pattern, fused with one another, or glands lined by, or papillae covered by, tumor cells demonstrating obvious piling-up. Poorly differentiated (p/d) carcinomas show mainly solid growth and only occasionally glandular/papillary patterns and/or mucus production. Blood vessel and lymphatic invasion was also explored microscopically, with hematoxylin-eosin and elastic-fiber stained sections of maximum tumour diameter made from paraffin-embedded specimens.

### SNP array analysis and comparisons of allelic imbalance at the chromosome arm level and in selected cancer-related regions

Extracted DNA was subjected to Affymetrix GeneChip Mapping 250K arrays. Allelic imbalance was analyzed using software termed the Copy Number Analyzer for Affymetrix Gene Chip Mapping (CNAG Ver. 2.0) [21]. After appropriate normalization of mean array intensities, signal ratios between tumours and anonymous normal references were calculated in an allele-specific manner, and allele-specific copy numbers were inferred from the observed signal ratios based on the hidden Markov model using the CNAG/AsCNAR software [21-23]. With this procedure, genomic profiles of *ALK* fusion-positive and -negative tumours were obtained. Datas have been deposited at NCBI's Gene Expression Omnibus data repository under GEO series accession number GSE41536.

Comparison was at two levels; a chromosome arm level and a smaller, specific gene locus level. To do this, first we compared average numbers of chromosome arms altered between the two groups [24]. We called gain or loss of each chromosomal arm when copy number change stretched more than 80% of entire length. Secondly, we compared recurrent copy number aberrations at twenty-one cancer-related loci with gains and five with losses. These specific regions were selected based on previous studies of the lung cancer genome [25,26] and through our global mapping with CNAG. The selected regions with relevant genes were as follows: for gains, 1p34.3 (*MYCL1*), 1q21.2 (*S100 family*), 3q29 (*MUC4*), 5p15.33 (*TERT*), 6p21.1 (*VEGF*), 7p11.2 (*EGFR*), 7p21.1, 7q31.2 (*MET*), 8q24.21 (*MYC*), 10q11.22, 12p12.1 (*KRAS*), 12q14.1 (*CDK4*), 12q15 (*MDM2*), 14q13.3 (*TTF1*), 16p13.3, 17q12 (*ERBB2*), 17q25.1, 19q12 (*CCNE1*), 20q13.2, 20q13.32, 20q13.33 (*TNFSF6B*); and for losses, 9p21.3 (*CDKN2A*), 9p23-p24.1 (*PTPRD*), 10q23.31 (*PTEN*), 13q14 (*RB1*), 17p13.1 (*TP53*).

### Statistical analysis

Clinicopathological parameters of cases with or without *ALK* fusion and the frequencies of chromosome arms changed and copy numbers of targeted loci were compared by the chi-square test or the Fisher’s exact test as appropriate. The average number of chromosome arms altered with or without *ALK* fusion was compared with Students’ *t*-test. Statistical significance was defined as *P*=0.05 or less.

## Results

### Comparisons of clinicopathological profiles of tumours with or without *ALK* fusion

Clinicopathological profiles of patients are summarized in Table 1. *ALK* fusion-positive cases were significantly younger and featured significantly more never-smokers (*P*=0.05, *P*=0.004, respectively). *ALK* fusion-positive tumours were histologically adenocarcinomas with notable characteristics such as poor differentiation as well as an acinar type structure and mucin production, as reported previously [7-9]. In this study, distribution of histological subtypes differed between two groups, namely, “acinar” subtype accounted for nearly forty percent in *ALK* fusion positive group (Table 1). The frequencies of vascular invasion, both of blood and lymph vessels, did not significantly differ between the two groups (*P*=0.738, *P*=0.273, respectively). In addition, the distribution of pathological stages did not vary (*P*=0.532).

**Table 1:** Comparison of clinicopathological parameters in cases with or without ALK fusion

	**with fusion**	**without fusion**	***P***
n	35	95	
Age (years)	58.5	62.8	0.050
gender			
male	14	47	0.337
female	21	48	
smoking			
never	25	41	0.004
ever	10	54	
pStage			
I	20	60	0.532
II-IV	15	35	
differentiation grade			
wel	4	44	<0.001
mod+por	31	51	
Predominant subtype			
papillary	21	77	0.019
Acinar	13	13	
Bronchioloalveolar	0	4	
solid with mucin	0	1	
signet	1	0	
lymphatic invasion			
-	24	68	0.738
+	11	27	
Vessel invasion			
-	15	51	0.273
+	20	44	
*TP53* mutation			
-	34	75	0.014
+	1	20	
*EGFR* mutation			
-	35	40	<0.0001
+	0	55	
*KRAS* mutation			
-	35	88	0.189
+	0	7	

### Mutational status of TP53, EGFR and KRAS

Data for the mutational status of *TP53, EGFR and KRAS* in the two groups are summarized in Table 1. Twenty-one cases had *TP53* mutations. Only one case with *ALK* fusion (Case 9: 1/35, 3%) harbored a mutation, a G/A transition at codon 273, as compared to 20 cases without *ALK* fusion (20/95, 21%), the mutation rates being significantly different (*P*=0.014) (Table 1, Additional file 1: Table S2). Twelve (12/21, 57%) of the *TP53*-mutated cases had a smoking history.

*EGFR* and *KRAS* mutations were not detected among *ALK* fusion-positive tumours. This fact that *ALK* rearrangement was mutually exclusive with *EGFR* and *KRAS* mutations (*P*<0.0001, *P*=0.189, respectively) is in line with our previous studies [8]. The *EGFR* mutation rate was 58% (55/95) in *ALK* fusion-negative cases and decreased with the smoking burden: 70.7% (29/41) in never smokers, 62.5% (15/24) in light smokers (0<pack-years<20) and 36.7% in heavy smokers (more than 20 pack-years) (11/30) (Additional file 2: Figure S1). *KRAS* mutations were identified in 7.4% (7/95) of *ALK* fusion-negative cases, and detected only among smokers. Though *KRAS* mutations were examined through codons 12, 13 and 61, they were found only in codon 12. The *KRAS* mutation rate increased along with the elevation of smoking index (Additional file 2: Figure S1). These findings for *EGFR* and *KRAS* mutations are consistent with previous reports from Japan, the prevalence being quite different from that in the United States [27-29].

### DNA copy number alterations of chromosome arms

**Figure 1:**
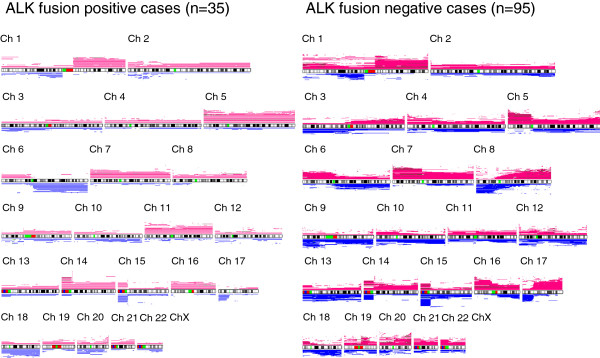
**Global view of copy number alterations with or without *ALK *fusion.** A line above a chromosome represents one case with genomic gain and its length. The color indicates the copy number of genomic regions: pink, 3 or 4 copies; and dark-red, ≥ 5 copies. Lines beneath the chromosomes represent copy number loss: blue, 1 copy; and light-green, 2 copies (homozygous deletion). Width between the lines of the two groups are adjusted according to the number of cases included for ease of visual comparison.

We compared the allelotypes of each chromosome arm between the two groups. Global views of chromosome aberrations are shown in Figure 1. Note that in *ALK* fusion-positive tumours, genomic copy number changes were more evenly distributed over the chromosome arms and high copy number gains (dark-red) in short genomic segments were less frequently encountered than with *ALK*-fusion negative examples. Significantly different patterns of respective chromosomal arm gain and loss were noted between the two groups. In fact, 5q, 8p, 9q, 11p and 11q were significantly more amplified, and 6q was more deleted in *ALK* fusion-positive tumours, whereas, in *ALK* fusion-negative tumours, 17q was more amplified, and 8p and 9p were more deleted (Figure 1, Table 2a, Additional file 1: Table S3-S5). *P*-values for comparisons of the aberration frequency in each chromosome arms are shown in Additional file 1: Table S5. When comparing global chromosome instabi-lity levels between the two groups, average numbers of chromosome arms with copy number gain or loss were 8.42 ±7.46 and 9.56 ±7.90 for tumours with and without *ALK* fusion, respectively, as detailed in Table 3, the difference not being statistically significant.

**Table 2:** **Comparisons of significantly altered chromosomal arms between adenocarcinomas with and without *ALK *fusion**

**Category**	**Gain**	**Loss**
More frequent *with* ALK fusion	5q, 8p, 9q, 11p, 11q	6q
More frequent *without* ALK fusion	17q	8p, 9q

**Table 3:** **Comparisons of numbers of chromosome arms with aberrations between adenocarcinomas with or without *ALK *fusion**

	**with *ALK *fusion (n=35)**	**without *ALK *fusion (n=95)**	***P***
Gains	5.97±6.75	6.21±6.95	0.859
Losses	2.46±3.06	3.35±4.34	0.196
Total	8.42±7.46	9.56±7.90	0.454

Note that significant differences are not detected.

### Chromosomal number alterations with advancement of pathological stage

**Figure 2:**
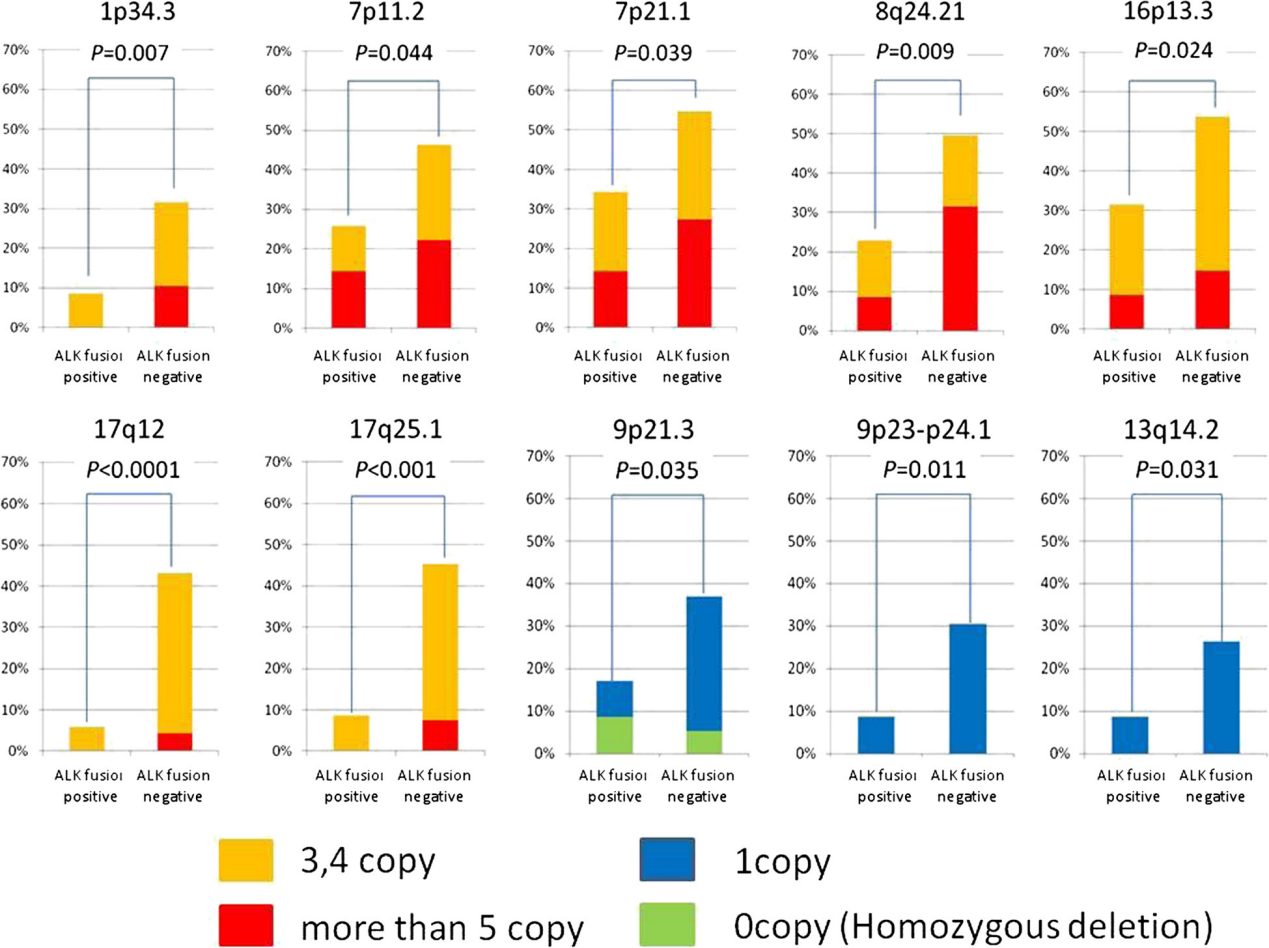
**Comparisons of numbers of chromosome arms altered with or without *ALK *fusion in different pathological stages.** Note that, whereas tumours in higher stages show more gains than stage I tumours when the tumours have no *ALK* fusion, *ALK* fusion positive tumours exhibit no such difference. p-Stage; pathological stage, n.s.; not statistically significant.

Chromosome aberration might be expected to increase as tumours progress in stages and, if so, numbers of chromosome arms with gain and/or loss might be larger in advanced tumours. In fact however, when we compared the number of chromosome arm altered between pathological stage I and II-IV, total number did not increase in pathological stage II-IV, though only *ALK* fusion-negative tumours showing significant elevation of chromosomal gain (Figure 2).

### Comparison of gain and loss frequency of selected loci

**Figure 3:**
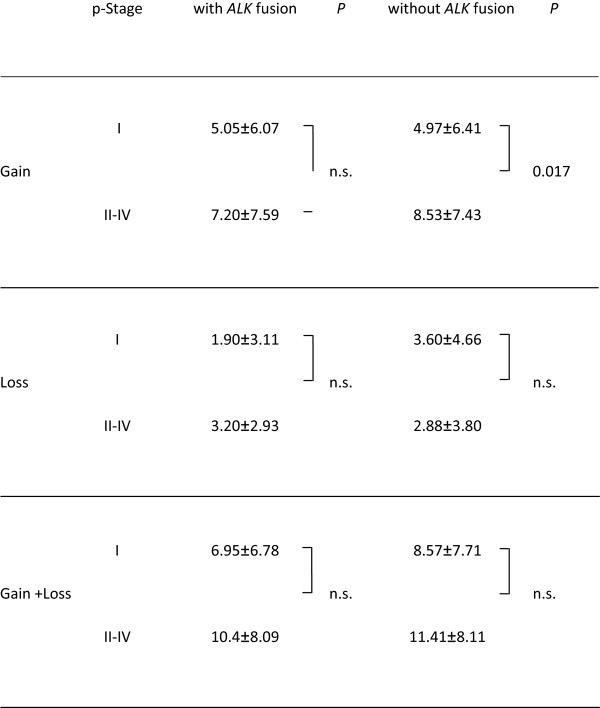
**Significant differences in copy number change detected at seven loci for gain and three loci for loss among twenty-six selected loci.** Colors of the stacked bars represent copy number: orange, 3,4 copies; red, ≥ 5 copies; blue, 1 copy loss (heterozygous deletion), light green: 2 copy losses (homozygous deletion). 17q12, 17q25.1 show remarkable differences in copy number gain between *ALK* fusion-positive and -negative tumours.

We selected twenty-one loci with recurrent copy number gain and five loci with loss to compare small-scale genomic aberrations. All the loci examined and *P*-values are summarized in Additional file 1: Table S6, S7. In Figure 3, stacked bar charts are shown indicating the percentage gain or loss of the selected loci. Interestingly, copy numbers (and related genes) at 1p34.3 (*MYCL1*), 7p11.2 (*EGFR*), 7p21.1, 8q24.21 (*MYC*), 16p13.3, 17q12 (*ERBB2*) and 17q25.1 were significantly less gained, and those at 9p21.3 (*CDKN2A*), 9p23-p24.1 (*PTPRD*), 13q14.2 (*RB1*) were significantly less deleted in *ALK* fusion-positive tumours than fusion-negative ones, with loci related to both oncogenes and tumour suppressor genes having fewer changes in tumours with *ALK* fusion. There were no oncogene-related loci with more gains and no suppressor gene-related loci with more losses in tumours with *ALK* fusion.

Homozygous deletions were found only at 9p21, at frequencies similar between the two groups, although the summed frequencies of heterozygous and homozygous deletions at 9p21.3 did significantly differ. In the group without *ALK* fusion, all the cases with the homozygous deletion harbored *EGFR* mutations.

*MYCL1, EGFR, MYC* and *ERBB2* are well-known oncogenes and *CDKN2A* and *RB1* are tumour suppressor genes related to lung carcinogenesis. *PTPRD* has been suggested to function as a tumour suppressor in several tumours, including lung cancers [30] and brain tumours [31]. Notably, 5p15.33, including *TERT* (telomerase reverse transcriptase), had the highest rate of gain in both groups regardless of *ALK* fusion (Additional file 3: Figure S2 and Additional file 1: Table S7).

Taken together, *ALK* fusion-positive tumours showed similar levels of overall chromosome instability, but when focusing on particular cancer-related regions, significantly fewer copy number gains at oncogene-related loci and significantly fewer deletions at suppressor gene-related loci.

## Discussion

Recurrent chromosome translocation has been accepted to play an important role in the pathogenesis of hematological malignancies, but not of solid tumours. Recently, however, chromosome rearrangements in solid tumours such as prostate cancer and non-small cell lung cancer have been reported [32]. *ALK* fusion was originally described in anaplastic large-cell lymphoma as a chimeric protein *NPM-ALK* resulting from a translocation. More recently, evidence has accumulated that the *EML4-ALK* fusion gene defines a novel subclass of lung adenocarcinomas with distinct clinicopathological features [7-9], so that it has emerged as a target for therapy. We focused here for the first time on allelic imbalance of tumours with *ALK* fusion with a novel technique which has already shown the involvement of loss of A20 function in the pathogenesis of a subset of B-cell lymphomas [33] and gain of function of C-CBL tumour suppressor in myeloid neoplasms [34]. Applying this methodology, we demonstrated that lung adenocarcnomas with *ALK* fusion feature less amplification of loci with oncogenes and fewer deletions of loci related to tumour suppressor genes, although global chromosome aberrations were similar between tumours with and without *ALK* fusion. suggesting that the fusion gene is a driver mutation, not just a passenger mutation.

Genetic instability was here categorized into two groups for simplicity, at the chromosomal level and at the nucleotide level. We earlier found the former to play a more important role in lung carcinogenesis, the frequency of LOH (loss of heterozygosity) being higher in less-differentiated tumours [35]. *ALK* fusion positive tumours are more common among non-smokers and the younger population, similar to those with *EGFR* mutations. We had expected fewer chromosome aberrations in *ALK* fusion-positive tumours because tumours arising in such people usually harbor less LOH and a lower *TP53* mutation rate than smokers [36-38]. Contrary to our expectation, the global copy number changes at the chromosomal arm level did not differ between the two groups, although significant differences of alteration frequency at the individual chromosomal arms were seen. In addition, only *ALK* fusion-negative tumours showed an increase of the frequency of chromosome arm gain with the advancement of disease stage. Furthermore, at the smaller-genomic scale level, *ALK* fusion-positive tumours were less amplified at the loci containing *EGFR* family genes, 7p11.2 (*EGFR*), 17q12 (*ERBB2*) and other loci, 1p34.3 (*MYCL*), 7p21.1, 8q24.21 (*MYC*), 16p13.3 and 17q25.1. *EGFR* and *ERBB2* play important roles by dimerizing when their ligands binds to produce downward growth signals to the tumour cells. Mutations and activation of these genes may drive carcinogenesis [39], and increased expression is associated with a poor prognosis in NSCLCs [40-43]. *ALK* fusion positive tumours are speculated to be less dependent on the actions of oncogenes and tumour-suppressor genes induced by copy number changes. Our results may also indicate that there is common and frequent chromosome abnormality in lung adenocarcinomas independent of *ALK* fusion, such as the 5p15.33 region, including *TERT.*

As for genomic loss, 9p21.3 (*CDKN2A*), 9p23-p24.1 (*PTPRD*) and 13q14.2 (*RB1*) were significantly less frequently deleted in *ALK* fusion-positive tumours. Homozygous deletion was seen only at 9p21.3 including *CDKN2A* and limited to *EGFR*-mutated tumours among *ALK* fusion-negative neoplasms as reported in the literature [44] and also seen in *ALK*-fusion positive ones*.* That deletion of 9p23-24.1 and 13q14.2 including tumour suppressor genes was rare in *ALK* fusion-positive tumours suggests that they can grow even if the functions of these suppressor genes are retained.

Of all the selected loci, 5p15.33 containing *TERT* (telomerase reverse transcriptase isoform 2) showed the highest frequency of recurring gain regardless of *ALK* fusion. The enzyme is important for telomere regeneration and maintenance resulting in a growth advantage and Zhang et al. reported that the locus is a frequent target of amplification during tumourigenesis [45]. Copy number gain of this locus significantly correlates with telomerase activity [46] and is one of the most consistent alterations in the early stages of non-small cell lung cancer [47]. In addition, increased susceptibility to lung cancer development associated with a SNP polymorphism of this locus has been reported [48,49]. The fact that most human tumour cells have telomerase activity indicates that its acquisition is vital for carcinogenesis and cell immortalization, and it might explain the reason why lung adenocarcinomas with or without *ALK* fusion shows similar frequency of copy number gain of this locus.

Our results have some therapeutic relevance. The fact that there are less involvement of other oncogenes and tumor suppressor genes may be related to dramatic responses to targeted drugs because of intact cellular processes including apoptosis pathways. In this regard, there is an interesting paper by Camidge et al. [50], demonstrating the inverse relationship between fused and isolated red copy number on FISH might suggest the *ALK* fusion positive tumor was a “near-diploid” subtype of non-small cell lung cancer. Comparing closely, however, between their and our results, our study clearly revealed the overall frequency of chromosome aberrations are similar between *ALK* fusion positive and negative tumors, suggesting not “near-diploid”. But, certainly, we need more investigations on genomic instability of *ALK* fusion positive tumors.

It is well known that smoking causes genomic changes with allelic imbalance [20]. As shown in Table 1, smokers dominate never smokers in the group without fusion whereas the fusion-positive group has more never smokers than smokers. Since the tumors without *ALK* fusion include *EGFR*-mutated tumors, most of which are from never smokers, the *ALK* fusion-negative group is certainly heterogeneous. In due course, a study that describes comparisons of allelotypes of non-smoker’s tumors between with *ALK* fusion and with *EGFR* mutation should be warranted.

## Conclusions

Although overall frequencies of aberrations at the chromosome arm level do not appear to significantly differ between *ALK* fusion-positive and -negative tumors, smaller genomic regions including cancer-related genes do show significant variation. Thus tumors with *ALK* fusion feature significantly fewer copy number gains and losses at loci containing oncogenes and tumor-suppressor genes, respectively. This implies that *ALK* fusion itself exerts very strong driving forces for tumorigenesis, in other words, that *ALK* fusion is a driver mutation, not just a passenger mutation.

## Competing interest

The authors have no potential conflicts of interest.

## Authors’ contributions

HN, MK, SO, HM and YI designed the study. HN, KT, KI, NM, HM and YI performed pathological and/or genomic diagnosis of tumors. HN, MK, MS and SO obtained microarray data and carried out bioinformatics analysis. HN and KN analyzed mutations. YS, SO and YI collected samples and/or provided detailed clinical data of patients. HN and YI drafted the manuscript. All authors read and approved the final manuscript.

## Authors’ information

HM has found ALK fusion in lung cancer with own developed cDNA library. MK, MS and SO detected genes responsible for hematological disorders through same algorithm with this study, CNAG/AsCAR. KT has created a novel diagnostic method to detect *ALK* fusion positive lung cancer. YI has found characteristic pathological features of *ALK* positive cancer.

## Pre-publication history

The pre-publication history for this paper can be accessed here:

http://www.biomedcentral.com/1471-2407/13/8/prepub

## Supplementary Material

Additional file 1: Table S1. Frequencies of fusion variants of *ALK* rearrangements. **Table S2.** Cases with *TP53* mutations and their smoking status. **Table S3.** Chromosomal arms and number of cases with gain with or without ALK fusion. **Table S4.** Chromosomal arms and number of cases with loss with or without ALK fusion. **Table S5.** P-values for comparisons of the frequencies of chromosome aberrations in all chromosome arms between tumours with or without *ALK* fusion. **Table S6.** Number of cases with copy number gain or loss at selected loci with or without *ALK* fusion. **Table S7.** Significance of the differences in frequencies of copy number changes (gains and losses) between tumours with or without *ALK* fusion. Click here for file

Addtional file 2: Figure S1. Mutation rates for EGFR, TP53 and KRAS according to cumulative smoking are shown. EGFR and KRAS mutations were only detected among ALK fusion negative cases, so ALK fusion positive cases were not included in the analysis. Note the gradually decrease in EGFR mutation rate with increase in cumulative smoking. KRAS mutations were detected only among smokers. Click here for file

Additional file 3: Figure S2 Comparisons of copy number alteration rates at selected loci with or without *ALK* fusion. Note that 5p15.33 including *TERT* shows the highest gain both in *ALK* fusion positive and negative tumours, the frequencies being identical. Click here for file
