# Supplementary material for: Allelotypes of lung adenocarcinomas featuring ALK fusion demonstrate fewer onco- and suppressor gene changes
Source: BMC Cancer. 2013 Jan 5;13:8. doi: 10.1186/1471-2407-13-8 (PMC3599044; doi:10.1186/1471-2407-13-8)
Supplement: Additional file 1: Table S1. — Frequencies of fusion variants of ALK rearrangements. Table S2. Cases with TP53 mutations and their smoking status. Table S3. Chromosomal arms and number of cases with gain with or without ALK fusion. Table S4. Chromosomal arms and number of cases with loss with or without ALK fusion. Table S5. P-values for comparisons of the frequencies of chromosome aberrations in all chromosome arms between tumours with or without ALK fusion. Table S6. Number of cases with copy number gain or loss at selected loci with or without ALK fusion. Table S7. Significance of the differences in frequencies of copy number changes (gains and losses) between tumours with or without ALK fusion. [file 1471-2407-13-8-S1.doc]

Supplementary Table S1. Frequencies of fusion variants of *ALK* rearrangements.

Variant fused exon n (%)

V1 E13;A20 9 26

V2 E20:A20 7 20

V3 E6;A20 11 31

V4 E15;A20 1 3

V5 E2;A20 1 3

V6 E13;A20 1 3

V7 E14;A20 1 3

KIF5B KIF5B;A20 2 6

N.A. 2 6

Total 35

N.A.: not analyzed

Supplementary Table S2. Cases with *TP53* mutations and their smoking status

No smoking status mutational pattern codon *ALK* fusion

1 never smoker transversion G→T 242 negative

2 never smoker transversion G→T 245 negative

3 never smoker transversion G→T 245 negative

4 never smoker transversion G→C 198 negative

5 never smoker transition G→A 175 negative

6 never smoker transition C→T 342 negative

7 never smoker transition A→G 179 negative

8 never smoker insertion 6bp exon 6 negative

9 never smoker transition G→A 273 positive

10 ever smoker transversion G→T 157 negative

11 ever smoker transversion T→G 215 negative

12 ever smoker transversion G→T 274 negative

13 ever smoker transversion G→T 337 negative

14 ever smoker transversion G→T 242 negative

15 ever smoker transition G→A 242 negative

16 ever smoker transition A→G 168 negative

17 ever smoker transition G→A 244 negative

18 ever smoker insertion 22bp exon 5 negative

19 ever smoker deletion 15bp exon 6 negative

20 ever smoker deletion 1bp exon 5 negative

21 ever smoker deletion 12bp exon 5 negative

Supplementary Table S3. Chromosomal arms and number of cases with gain with or without ALK fusion.

ALK fusion ALK fusion

positive negative positive negative

　　　　　　　　　　n　　　　　　　　　　　　　　　　　　　　n

1p 1 8 11p 10 10

1q 14 37 11q 11 10

2p 3 13 12p 2 10

2q 7 12 12q 2 6

3p 2 3 13q 4 6

3q 3 15 14q 9 18

4p 4 20 15q 1 3

4q 4 10 16p 9 39

5p 17 42 16q 9 19

5q 14 18 17p 0 6

6p 6 27 17q 0 36

6q 0 6 18p 5 14

7p 13 47 18q 2 2

7q 10 30 19p 5 12

8p 5 1 19q 4 14

8q 5 24 20p 3 15

9p 2 2 20q 4 22

9q 4 2 21q 5 17

10p 3 12 22q 2 12

10q 1 4

Supplementary Table S4. Chromosomal arms and number of cases with loss with or without ALK fusion.

ALK fusion ALK fusion

positive negative positive negative

　　　　　　　　　 n　　　　　　　　　　　　　　　　　　n

1p 1 0 11p 1 8

1q 0 0 11q 1 6

2p 1 5 12p 2 13

2q 1 6 12q 1 9

3p 2 15 13q 3 10

3q 2 4 14q 1 2

4p 1 5 15q 3 11

4q 1 12 16p 0 1

5p 1 0 16q 1 13

5q 1 4 17q 10 26

6p 2 2 17q 0 0

6q 12 12 18p 8 11

7p 1 0 18q 9 16

7q 1 4 19p 2 7

8p 1 26 19q 1 7

8q 0 3 20p 7 7

9p 1 17 20q 1 1

9q 2 19 21q 0 4

10p 2 13 22q 2 5

10q 4 16

Supplementary Table S5. P-values for comparisons of the frequencies of chromosome aberrations in all chromosome arms between tumours with or without *ALK* fusion.

Gain Loss Gain Loss

1p 0.4430 0.2692 11p 0.0114 0.4430

1q 0.9132 1.0000 11q 0.0041 0.6737

2p 0.5559 1.0000 12p 0.5123 0.3525

2q 0.4005 0.6737 12q 1.0000 0.2862

3p 0.6107 0.1549 13q 0.4570 1.0000

3q 0.3959 0.6599 14q 0.4659 1.0000

4p 0.3083 1.0000 15q 1.0000 0.7580

4q 1.0000 0.1836 16p 0.1511 1.0000

5p 0.6578 0.2692 16q 0.4798 0.1107

5q 0.0134 1.0000 17p 0.1904 1.0000

6p 0.2569 0.2932 17q 0.0000 1.0000

6q 0.1904 0.0093 18p 1.0000 0.1588

7p 0.2110 0.2692 18q 0.2932 0.3159

7q 0.7417 1.0000 19p 0.7758 1.0000

8p 0.0054 0.0013 19q 0.7785 0.6816

8q 0.2376 0.5632 20p 0.3959 0.0549

9p 0.2932 0.0414 20q 0.2153 0.4675

9q 0.0446 0.0606 21q 0.7938 0.5735

10p 0.7582 0.3525 22q 0.3497 1.0000

10q 1.0000 0.5877

Supplementary Table S6. Number of cases with copy number gain or loss at selected loci with or without *ALK* fusion

GAIN LOSS

*ALK* fusion *ALK* fusion

locus positive negative locus positive negative

1p34.3 3 30 9p21.3 6 35

1q21.2 13 49 9p23-24.1 3 29

3q29 4 26 10q23.31 5 18

5p15.33 22 58 13q14.2 3 25

6p21.1 6 34 17p13.1 10 28

7p11.2 9 44

7p21.1 12 52

7q31.2 11 29

8q24.21 8 47

10q11.22 2 17

12p12.1 5 20

12q14.1 8 17

12q15 8 19

14q13.3 10 29

16p13.3 11 51

17q12 2 41

17q25.1 3 43

19q12 5 26

20q13.2 7 29

20q13.32 7 37

20q13.33 11 45

Supplementary Table S7. Significance of the differences in frequencies of copy number changes (gains and losses) between tumours with or without *ALK* fusion.

Selected loci with cancer related Selected loci with cancer-related

copy number gain *P* gene copy number loss *P* gene

1p34.3 0.007 MYCL 9p21.3 0.035 CDKN2A

1q21.2 n.s. S100 9p23-24.1 0.011 PTPRD

3q29 n.s. 10q23.31 n.s. PTEN

5p15.33 n.s. TERT 13q14.2 0.031 RB1

6p21.1 n.s. 17p13.1 n.s. TP53

7p11.2 0.044 EGFR

7p21.1 0.048

7q31.2 n.s. MET

8q24.21 0.009 MYC

10q11.22 n.s.

12p.12.1 n.s. KRAS

12q14.1 n.s. CDK4

12q15 n.s. MDM2

14q13.3 n.s. TITF1

16p13.3 0.002

17q12 <0.0001 ERBB2

17q25.1 <0.001

19q12 n.s. CCNE1

20q13.2 n.s.

20q13.32 n.s.

20q13.33 n.s.
