# Supplementary figures and images for: Allelotypes of lung adenocarcinomas featuring ALK fusion demonstrate fewer onco- and suppressor gene changes
Source: BMC Cancer. 2013 Jan 5;13:8. doi: 10.1186/1471-2407-13-8 (PMC3599044; doi:10.1186/1471-2407-13-8)

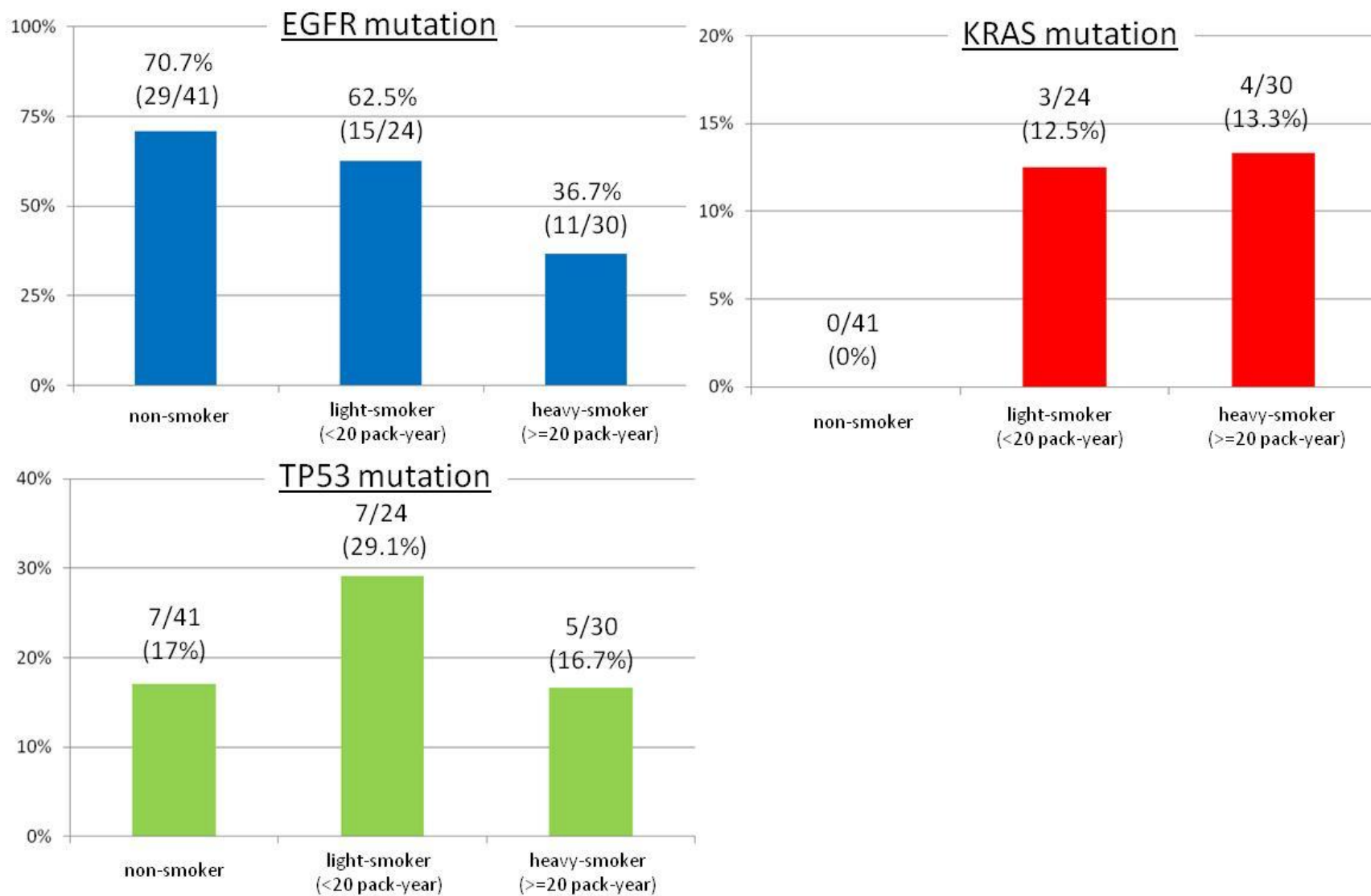

Ninomiya et al. Supplementary Fig S1.

Supplement: Addtional file 2: Figure S1. — Mutation rates for EGFR, TP53 and KRAS according to cumulative smoking are shown. EGFR and KRAS mutations were only detected among ALK fusion negative cases, so ALK fusion positive cases were not included in the analysis. Note the gradually decrease in EGFR mutation rate with increase in cumulative smoking. KRAS mutations were detected only among smokers. [file 1471-2407-13-8-S2.pdf]

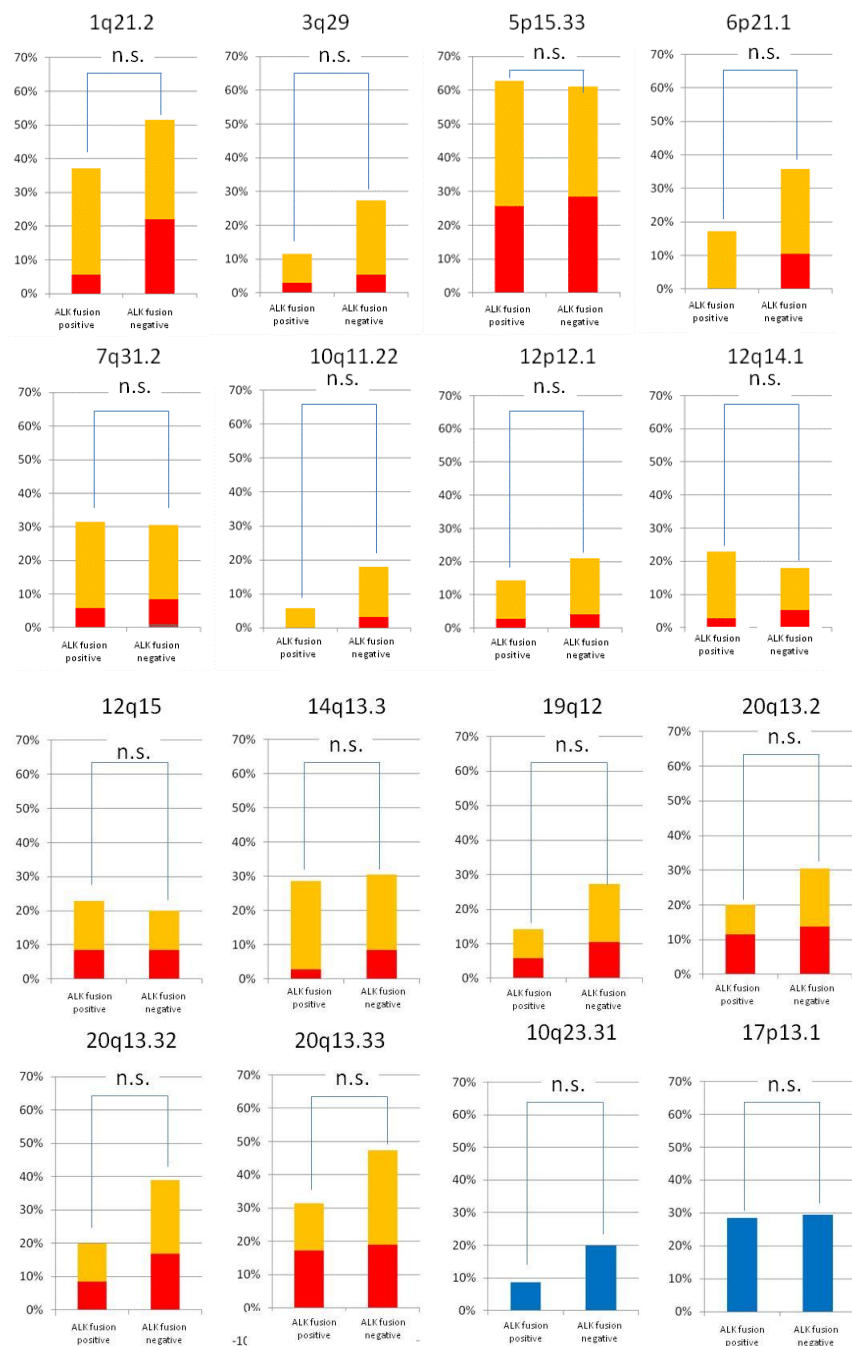

Supplement: Additional file 3: Figure S2 — Comparisons of copy number alteration rates at selected loci with or without ALK fusion. Note that 5p15.33 including TERT shows the highest gain both in ALK fusion positive and negative tumours, the frequencies being identical. [file 1471-2407-13-8-S3.pdf]
